# Tissue Distribution and Anti-Lung Cancer Effect of 10-Hydroxycamptothecin Combined with Platycodonis Radix and Glycyrrhizae Radix ET Rhizoma

**DOI:** 10.3390/molecules24112068

**Published:** 2019-05-30

**Authors:** Wugang Zhang, Mulan Li, Wendi Du, Wuliang Yang, Guofeng Li, Chen Zhang, Xinli Liang, Haifang Chen

**Affiliations:** 1 National Pharmaceutical Engineering Center for Solid Preparation in Chinese Herb Medicine, Jiangxi University of Traditional Chinese Medicine, Nanchang 330004, China; zwgchf98@126.com (W.Z.); 15570351171@163.com (M.L.); wendi_du_0216@163.com (W.D.); 18379973507@163.com (G.L.); 18363259880@163.com (C.Z.); 2 Key Laboratory of Modern Preparation of Traditional Chinese Medicine, Ministry of Education, Jiangxi University of Traditional Chinese Medicine, Nanchang 330004, China; yangwuliang@163.com

**Keywords:** 10-hydroxycamptothecin, Platycodonis Radix, Glycyrrhizae Radix ET Rhizoma, tissue distribution, anti-lung cancer efficacy

## Abstract

10-Hydroxycamptothecin (HCPT) is a broad-spectrum chemotherapeutic drug, although its side effects and multidrug resistance (MDR) limit its clinical application. A range of drug delivery systems have been utilized to overcome its shortcomings and maintain its therapeutic efficacy, however the use of the transport effect of traditional Chinese medicines (TCMs) to improve the distribution of chemotherapeutic drugs has not been widely reported. Platycodonis Radix (JG) and Glycyrrhizae Radix ET Rhizoma (GC) are common TCMs in clinics and are often combined as drug pairs to act as “transport agents”. In the present study, the effect of JG and GC (JGGC) on the distribution of HCPT in tissues and its antitumor efficacy after being combined as a therapy were investigated, for which ultrahigh-performance liquid chromatography-tandem mass spectrometry (UHPLC-MS/MS) was used. Furthermore, the effect on the protein expression of multidrug resistance proteins (P-gp and LRP), and the immunomodulatory and synergistic antiapoptotic effect on Lewis lung cancer-bearing C57BL/6J mice were also evaluated. The results demonstrate that JGGC significantly increased the area under the concentration time curve (AUC) and mean residence time (MRT) and reduced the clearance rate (CL) of HCPT. In addition, the combined use of JGGC decreased the levels of LRP, P-gp and Bcl-2/Bax when treated with HCPT. JGGC also significantly elevated the levels of RBCs, PLTs, HGB, IL-2, and IFN-γ, and decreased IL-10 levels. In summary, an increased concentration of HCPT in tissues was observed when it was combined with JGGC through inhibition of efflux protein, with a synergistic enhancement of the anticancer effect observed through promotion of apoptosis and immunity due to a reversion of the Th1/Th2 shift. Our findings provide a reference for the feasibility of combining JGGC with chemotherapy drugs in clinical applications.

## 1. Introduction

10-Hydroxycamptothecin (HCPT) is an indole alkaloid that was isolated from *Camptotheca acuminate* in the 1960s [1]. HCPT exhibits antitumor activity against a wide spectrum of human malignancies whose mechanism is related to the selective inhibition of topoisomerase I which interferes with DNA replication [2]. Due to its low solubility, unstable lactone ring and the expression in tumors of multidrug resistance-related proteins, the bioavailability of HCPT is reduced, which greatly limits its concentration and efficacy at target sites [3,4,5]. Therefore, increasing the concentration of HCPT at the sites of lesions is often the focus of cancer researchers. Many investigators have explored high-performance drug delivery systems such as nanoparticles [6], micelles [7], liposomes [8], and microspheres [9] to improve the bioavailability and efficacy of HCPT; however, most new delivery systems lack sufficient safety [10] and are still far from clinical application. Gradually, researchers are focusing their attention on combining chemotherapeutic drugs with traditional Chinese medicines to improve their bioavailability and targeting. At present, there are only a few reports of such combination therapies, such as *Radix astragali* and *Curcuma longa*, being able to improve the anticancer effect of HCPT [11].

Platycodonis Radix (JG), the root of *Platycodon grandiflorus* (Jacq.) A.DC., used in food in addition to medicine, is commonly used as a lung meridian medicine and in combination with other TCMs in the treatment of tumors in clinics [12,13]. Glycyrrhizae Radix ET Rhizoma (GC), the root of *Glycyrrhiza uralensis* Fisch., is commonly administered with other drugs due to its ability to synergistically enhance their effectiveness and reduce their toxicity, in addition to improving flavor in foods. JG and GC are often used as a drug pair to act as a transport agent. It has been listed in the “Origin of Medicine” compiled by Yuansu Zhang. JG and GC have been used in numerous formulations for the treatment of pulmonary neoplasms [14,15]. However, the effects of JG and GC in these formulations have rarely been investigated in depth. Furthermore, recent research has shown that a combination of JG or GC with other drugs increases the plasma concentration and tissue distribution of the medicinal ingredients with which they are combined [16,17].

Therefore, in the present manuscript, based on the transport effect of JG and GC, it is hypothesized that the combination of JG and GC (JGGC) with chemotherapeutic drugs could improve drug accumulation in tissue and has a synergistic antitumor effect. Firstly, the transport effect of JG and GC on the distribution of HCPT in tissue was investigated, in addition to the potential mechanisms of the modulation of drug resistance-related proteins (p-gp and LRP) by JGGC. Secondly, the synergistic anticancer effect of JGGC on immune function was also explored.

## 2. Results and Discussions

### 2.1. UHPLC-ESI MS/MS Method Validation

**Figure 1:**
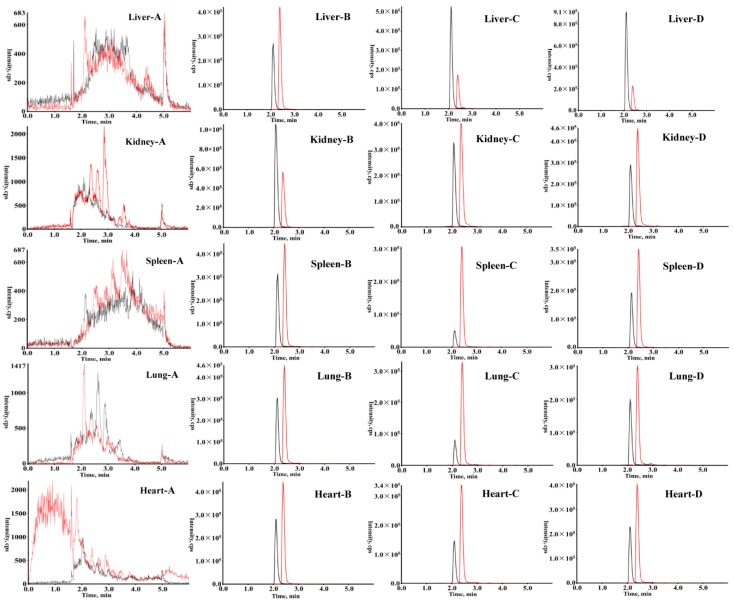
Chromatograms of HCPT and CPT: Blank tissue (**A**). Blank tissue spiked with HCPT and CPT (**B**). Tissue sample after intravenous administration of HCPT at 60 min (**C**). Tissue sample after administration of HCPT and JGGC extract at 60 min (**D**).

The analytes HCPT and camptothecin (CPT) appeared well-separated with no significant interference from endogenous substances, with retention times of 2.09 and 2.48 min for HCPT and CPT in MS conditions, respectively. Typical HCPT chromatograms displaying blank mouse tissue, blank mouse tissue spiked with HCPT and CPT, and experimental tissue samples are shown in Figure 1. As shown in Table 1, all HCPT-calibrated media exhibited good linearity (r > 0.9965). Specificity, precision and accuracy, recovery, and stability were analyzed. As shown in Table 2, the intraday and interday accuracy (Relative Error, RE) ranged from −13.4% to approximately 11.2% and −6.0% to 11.45% in biological samples, respectively. The relative standard deviation (RSD) in intraday and interday precision values was in the range of 1.07% to 9.58%. The results demonstrate that precision and accuracy values were within acceptable criteria. The extraction recovery for HCPT at different concentration levels ranged from 77.44% to 101.44%. Stability of samples was also investigated with freeze-thaw, short-term, and long-term storage, details of which are presented in Table 3. Together, the results above indicate that the method was consistent, precise, and reproducible, satisfying the analysis requirements for biological samples.

### 2.2. Tissue Distribution Study

The pharmacokinetic parameters in tissue, including half-life (T_1/2_), peak concentration (C_max_), area under the concentration-time curve (AUC_(0–t)_), mean residence time (MRT), clearance rate (CLz), and t_1/2_ (elimination half-life), were calculated using DAS 3.3.0 software, in accordance with a noncompartment model.

**Figure 2:**
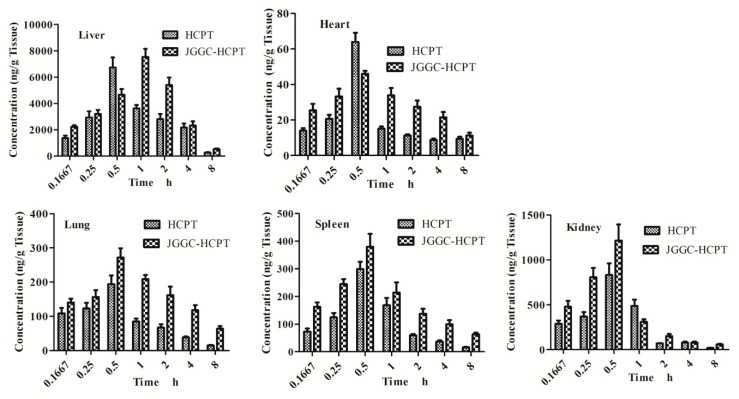
Effect of pretreatment with Platycodonis Radix-Glycyrrhizae Radix ET Rhizoma extract on primary tissue distributions of HCPT in C57BL/6J mice. Data are shown as means ± SD, n = 6.

After intravenous administration, HCPT rapidly accumulated in the liver, followed by the kidneys, spleen, lungs and, to the least extent, the heart. The tissue distribution of HCPT and pharmacokinetic parameters are presented in Figure 2 and Table 4. Except for the kidneys, the AUC and MRT of HCPT in the liver, spleen, lungs and heart increased when HCPT was combined with JGGC, while CLz decreased significantly (*p* < 0.05, *p* < 0.01), indicating that JGGC enhanced the accumulation of HCPT.

### 2.3. Effect of JGGC and HCPT on the Growth of Tumors in a Lewis Lung Carcinoma Mouse Model.

**Figure 3:**
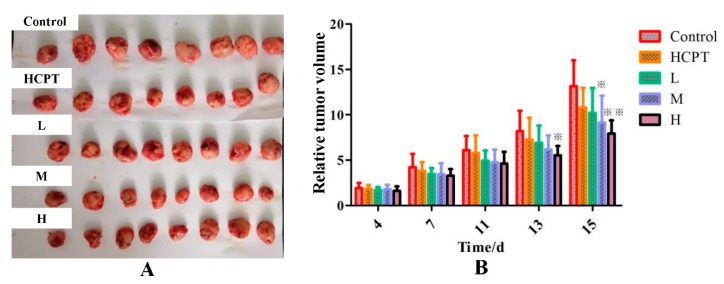
Effect of single or combination of JGGC with HCPT on the tumor growth of Lewis lung carcinoma mice. The photo of tumor collected from different groups of mice at the end of treatment (day 16) (**A**). Relative tumor volume of different groups of mice (**B**). Data are shown as mean ± SD, n = 10. ^※^ *p* < 0.05, ^※※^ *p* < 0.01 vs. control. L represents low dose group [HCPT (3.9 mg/kg) + JGGC (1.3 g/kg)], M represents medium dose group [HCPT (3.9 mg/kg) + JGGC (2.6 g/kg)], and H represents high dose group [HCPT (3.9 mg/kg) + JGGC (5.2 g/kg)].

As shown in Figure 3, the volume of the tumors in mice increased steadily after injection of Lewis lung carcinoma cells, with the growth rate of the tumors accelerating, especially in the later stages. During administration of the drug, although tumor growth continued to progress, the growth rate slowed after 11 days of administration. Compared with the control group, the relative volumes of tumors in the JGGC and HCPT groups were smaller. After a further two days, the benefits of combination therapy gradually emerged. The rate of tumor growth in the combined group was significantly slower than that of the control group (*p* < 0.05, *p* < 0.01). The results demonstrated that both single and combined therapy exhibited an antitumor effect, but combined administration was more dramatic. On the final day of the experiment, the tumors were weighed to calculate rates of inhibition caused by the therapy. The inhibition rate of HCPT alone was 33.83%, but was 42.97% for the medium dose of the combination therapy and 50.46% for the high dose. As shown in Table 5, the relative weights of the heart, lungs and kidneys were the same in the combination group as they were with HCPT alone. However, the relative weights of the livers in the control group increased significantly compared with that of the normal group, and the weights of the livers in the HCPT group were similar to those of the control group. Conversely, the relative weights of livers (%) of mice treated with a combination of JGGC and HCPT tended to be consistent with the livers of the normal group. Similarly, spleen hypertrophy was observed in tumor-bearing mice. After the treatment, no significant difference was observed in relative spleen weight (%) between the combination group and HCPT alone. These results indicate that the treatment with JGGC in the combination therapy could not cause or increase organ toxicity.

### 2.4. JGGC Enhanced the Chemotoxicity of HCPT Through Inhibition of Protein Expression of LRP and P-gp

Drug resistance to chemotherapy remains a major challenge in the treatment of cancer. Resistance-related proteins involved in lung cancer include P-gp and LRP [18,19,20]. After long-term chemotherapy, the increased expression of MDR-related protein leads to a reduction in the concentration of chemotherapeutic drug at the site of a lesion and failure of the therapy. Inhibition of P-gp and LRP is an important strategy to promote the accumulation of chemotherapeutic drugs in malignant cells and tissues.

The meridians of JGGC can promote the distribution of drugs into tissues, so we speculated whether the distribution of JGGC promoting drugs in tissues was related to inhibition of the expression of multidrug resistance protein. To further elucidate the mechanism of the increased distribution of HCPT into tissues after combination therapy, the effect of JGGC on the efflux of protein in mice bearing Lewis lung cancer was investigated.

**Figure 4:**
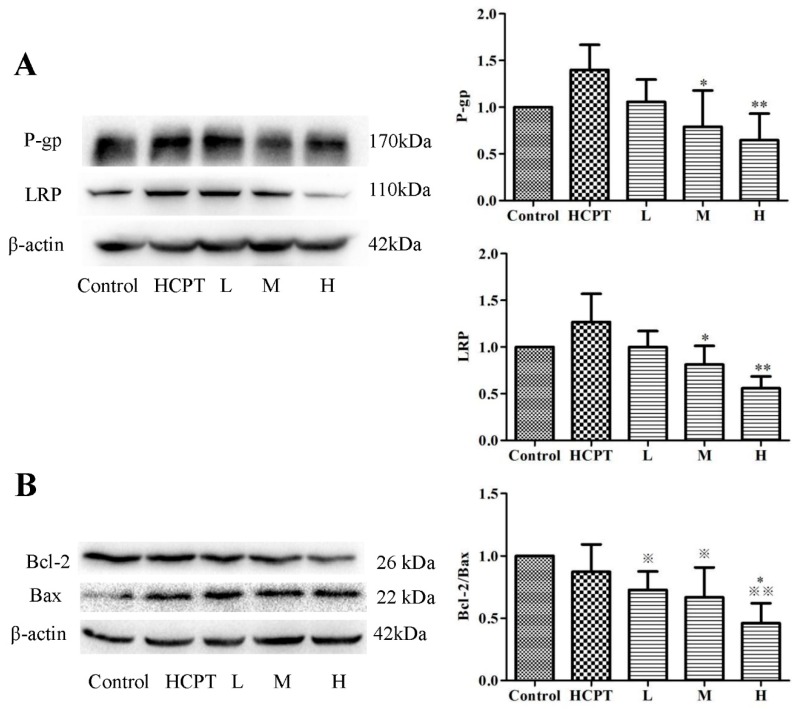
Effect of combination of JGGC on the protein express of LRP and P-gp (**A**) and the protein express of Bcl-2 and Bax (**B**). Data are shown as mean ± SD, n = 3. ^※^ *p* < 0.05, ^※※^ *p* < 0.01 vs. control; * *p* < 0.05, ** *p* < 0.01, vs. HCPT.

The results demonstrate that the protein expression of LRP and P-gp increased significantly in the control group, decreasing after the addition of JGGC, indicating that JGGC enhanced the distribution of HCPT into tissues through inhibition on LRP and P-gp. The effect of JG and GC on P-gp is consistent with previous reports [21,22]. The results are shown in Figure 4A.

### 2.5. JGGC Enhanced the Chemotoxicity of HCPT Through promotion of Apoptosis

Numerous MDR-linked genes including not only ATP-binding cassette (ABC) transporters, but also Bcl-2 family genes, are involved in MDR. Loss of apoptotic genes or overexpression of antiapoptotic genes will lead to drug resistance in cancer cells. Bcl-2 is a family of proteins that regulate apoptosis by inducing the expression of proapoptotic proteins or inhibition of the expression of antiapoptotic proteins. Bcl-2 is considered an important antiapoptotic protein in the Bcl-2 family, which has a protective effect on tumor cells and is conducive to the survival and growth of tumors. Bax, on the other hand, promotes apoptosis. Targeting the Bcl-2 family proteins could be a suitable approach to resolve MDR [23]. Our results show that Bcl-2 expression is significantly increased and Bax decreased in the control group, while therapy with JGGC synergistically inhibited the protein expression of Bcl-2 while increasing protein expression of Bax(*p* < 0.01, *p* < 0.05). The results are shown in Figure 4B and suggest that JGGC further promotes cell apoptosis and plays a synergistic role in the antitumor effect.

### 2.6. Effect of the Combination of JGGC and HCPT on Blood Parameters in Lewis Lung Carcinoma Mice

**Figure 5:**
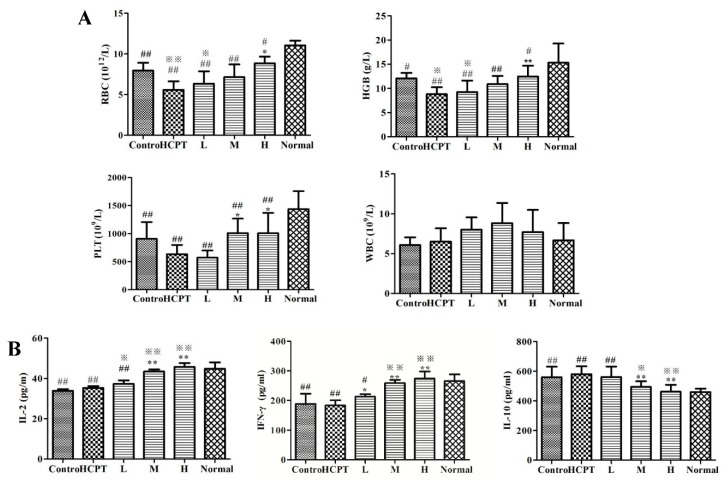
Combination of JGGC and HCPT on the routine blood (WBCs, RBCs, HGB, and PLTs) (**A**) and immune cytokine (IL-2, IL-10, and IFN-γ) (**B**) of Lewis lung carcinoma mice. Data are shown as mean ± SD, n = 10. ^#^ *p* < 0.05, ^##^ *p* < 0.01 vs. normal; ^※^ *p* < 0.05, ^※※^ *p* < 0.01 vs. control; * *p* < 0.05, ** *p* < 0.01, vs. HCPT.

Emerging evidence has demonstrated that when TCMs are combined with chemotherapeutic drugs the quality of life and survival rate of patients improve [14]. However, the mechanisms involved are unclear. While chemotherapeutic drugs are administered as a treatment for tumors, bone marrow and hematopoietic dysfunction, they worsen the immunity of patients. Therefore, in this study, we wished to investigate whether treatment with JG and GC improved the immunity of mice. Compared with the normal group, the number of white blood cells (WBCs) was not significantly different, but the number of red blood cells (RBCs), hemoglobin (HGB), and platelets (PLTs) in the control group decreased significantly (*p* < 0.01, *p* < 0.05). After treatment with HCPT, RBCs and HGB levels declined significantly compared with those of the control group (*p* < 0.05). However, after combined therapy with JGGC and HCPT, the levels of RBCs, HGB, and PLTs increased gradually compared with the single chemotherapy group, particularly in high dose group (*p* < 0.05, *p* < 0.01), as shown in Figure 5A.

### 2.7. Effect of Combination of JGGC and HCPT on IL-2, IL-10, and IFN-γ Levels in Lewis Lung Carcinoma Mice

Type 1 T-helper cells (Th1) produce IL-2 and IFN-γ, which are responsible for cell-mediated immunity against tumors. Conversely, Th2 cells, which predominantly secrete IL-4, IL-6, and IL-10, are involved in the regulation of humoral immune responses and antibody production. The balance of Th1 and Th2 cells plays an important role in regulating the immune response in tumor microenvironments [24,25]. Previous studies have reported that Th1 responses are suppressed and those of Th2 elevated systemically in lung cancer [26]. Similarly, the hematopoietic function and immunity of mice transplanted with Lewis lung cancer cells were further impaired after chemotherapy.

As shown in Figure 5B, the levels of the cytokines IL-2 and IFN-γ decreased significantly, while IL-10 increased in the control. After chemotherapy, the levels of these cytokines did not change, but combination therapy caused a significant rise in the levels of IL-2 and IFN-γ, and decreased the level of IL-10. Therefore, the observations that confirm the synergistic antitumor effect of JGGC could be related to improvements in the immune and hematopoietic function of the mice.

## 3. Materials and Methods

### 3.1. Chemicals and Reagents

Camptothecin (CPT) and HCPT were purchased from Chengdu Must Bio-Technology Co. Ltd. (Chengdu, China). HCPT injection was purchased from Harbin Sanlian Pharmaceutical Limited (Harbin, China). HPLC-grade acetonitrile (ACN) and methanol (MeOH) were obtained from Tedia Company Inc (Fairfield, CT, USA). HPLC-grade formic acid was purchased from Aladdin Co Ltd. (Shanghai, China). Purified water was prepared using a Milli-Q water purification system (Millipore, Bedford, MA, USA).

### 3.2. Preparation of JGGC Extract

JG and GC were mixed at a ratio of 1:1 and decocted twice with water (1:10, W/V) for 1 h. The two decoctions were then filtered and the water evaporated to obtain a JGGC extract (JGGC). The concentrations of platycodin D, liquiritin, glycyrrhizic acid, and glycyrrhizic acid ammonium salt in the JGGC were found to be 0.16%, 2.56%, 2.25%, and 2.3%, respectively, by HPLC analysis.

### 3.3. In Vivo Tissue Distribution

The effect of JGGC on the accumulation and distribution of HCPT was investigated in vivo. All experimental animal procedures and treatments were conducted strictly in accordance with the Guide for the Care and Use of Laboratory Animals, and approved by the Experimental Animal Ethics Committee of the Jiangxi University of Traditional Chinese Medicine (Permit No. SYXK (Gai)-2017-0004). Male C57BL/6J mice (20 ± 2 g) were obtained from the Laboratory Animal Center of Hunan Silaikejingda Experimental Animal Co. Ltd. All animals were housed in an environmentally-controlled room at 25 °C with a 12 h light/dark cycle for at least one week before the start of experimentation. Prior to each experiment, the mice were fasted for 12 h with free access to water. In the tissue distribution study, 84 mice were randomly assigned to either an HCPT or JGGC-HCPT group. The HCPT group received 3.9 mg/kg HCPT intravenously by tail vein injection, while the JGGC-HCPT group received HCPT intravenously 2 h after JGGC.

Extract gavage (2.6 g/Kg, equivalent to 2.6 g of crude drug). Tissue was harvested after 0.1667, 0.25, 0.5, 1, 2, 4, or 8 h (n = 6 for each time period). After desanguination, tissue samples were added into three times their volume of 0.9% NaCl solution and homogenized. Tissue homogenates were then stored at −20 °C until required for analysis.

### 3.4. In Vivo Analysis of HCPT Using UHPLC-ESI MS/MS

One-hundred microliters of tissue homogenate, 40 μL 0.1% formic acid, and 10 μL of CPT as internal standard were added, followed by 800 μL of acetonitrile. The resultant mixture was thoroughly vortex-mixed for 2 min, then centrifuged at 14,000 ×*g* for 10 min. The supernatant (600 μL) was collected and evaporated until dry under a gentle stream of nitrogen. The residue was reconstituted with methanol, then 2 μL injected into the LC-MS system for analysis.

Separation and quantification of the analytes was performed on an Agilent Extend-C18 column (2.1 mm × 100 mm, 3.5 μm) using UHPLC (Shimadzu, Kyoto, Japan) connected to an AB TRIPLE QUDA 5500 mass spectrometry system (AB SCIEX, Foster city, CA, USA). The solvents 0.1% formic acid (A) and ACN (B) were selected as the mobile phase. A gradient was programmed as follows; 0–3 min: 5%–90% B; 3–4 min: 90% B; 4.0–4.1 min: 90%–5% B; and 4.1–6 min: 5% B. A flow rate of 0.4 mL/min was maintained using a 2 μL injection volume. The ion source temperature was maintained at 550 °C with anion spray voltage of 5500 V. The curtain gas, collision gas, entrance, and collision exit potentials were set to 35 psi, 7 psi, 10 V, and 14 V, respectively. The declustering potentials for HCPT and CPT were 110 and 100 V, respectively. The collision energy was 31 eV for HCPT and CPT. The ion pairs used for quantitative analysis were 365.1 → 321.1 (HCPT) and 349.2 → 305.1 (CPT) in positive mode.

### 3.5. Preparation of Standard Solutions and Quality Control (QC) Samples

Stock solutions of HCPT and CPT were prepared in methanol. All solutions were stored at −20 °C. By spiking HCPT and/or CPT working standard solutions (10 μL) into specified volumes of tissue homogenate, a calibration standard curve was created. The final concentrations of the standards were in the range of 1 to 2000 ng/mL of tissue. The calibration curves were based on the relationship between the ratio of the peak area of HCPT to that of CPT, with data that was obtained with linear regression weighted by 1/x^2^. Quality control (QC) sample concentrations were listed in Table 2. Method validation, including selectivity, intraday accuracy and precision, and recovery, was also investigated according to the guidelines for analysis of biological samples.

### 3.6. Establishing Transplanted Lewis Lung Carcinoma Mouse Model.

Lewis lung cancer cells in the logarithmic growth phase were digested from culture, counted and prepared into suspensions of 5 × 10^6^/mL. 1 × 10^6^ cells were injected subcutaneously into the scapula of the right forearm of each mouse. After 10 days, the size of each tumor was measured. Mice with tumors of a uniform size and diameter of approximately 0.5 cm × 0.5 cm were selected for use in the experimental study.

### 3.7. Antitumor Effect of Combination of HCPT and JGGC Treatment

Tumor-bearing mice were randomly divided into five groups: model group (control), HCPT group (3.9 mg/kg), low dose group [HCPT (3.9 mg/kg) + JGGC (1.3 g/kg), L], medium dose group [HCPT (3.9 mg/kg) + JGGC (2.6 g/kg), M], and high dose group [HCPT (3.9 mg/kg) + JGGC (5.2 g/kg), H]. HCPT was injected every other day and JGGC administered by gavage daily for 15 days. Control group is with tumor cells but no therapy. Normal group was nontumor-bearing C57BL/6J mice. After sacrificing the animals, blood was collected and the tumors and organs weighed. Changes in tumor volume (TV) in the mice were recorded until the day before the end of the experiment. The width (W) and length (L) of each tumor were measured using a vernier caliper, with tumor volume calculated according to the following formula; TV (mm^3^) = (LW^2^)/2. The tumor growth inhibition rate was calculated using the formula IR (%) = (1−W/Wc) ×100, where W and Wc represent the mean weight of tumors in the treated and control groups, respectively. Relative tumor growth volume (RTV) was calculated using the formula RTV = V_t_/V_0_, where V_t_ and V_0_ represent the volumes of tumors after and before administration of therapy, respectively. The relative organ weights of the mice were measured to investigate toxicity caused by the combination of JGGC during the in vivo antitumor evaluation. Relative heart, liver, spleen, lung, and kidney weight were calculated using the following formula: Relative organ weight (%) = (mean organ weight/mean body weight) × 100.

### 3.8. Immunoregulation Effect of JGGC on HCPT Therapy

To assess the immune response after treatment with the combination of JGGC and HCPT, routine blood analysis was conducted using a Sysmex XT2000i automatic blood cell analyzer.

IL-2 was obtained from Boster Biological Technology Co. Ltd. IFN-γ and IL-10 were measured using MultiScience ELISA kits. The concentrations of cytokines in serum were measured at a wavelength of 450/570nm (BioTek Instruments, Inc., Winooski, VT, USA).

### 3.9. Western Blotting

Tumor tissue was lysed with RIPA lysing buffer then protein concentration established with a BCA kit. Equal quantities of tumor tissue lysate (30 μg protein) were separated by 12% SDS-PAGE, followed by Western blot analysis following transfer of the separated proteins to PVDF membranes. After incubation in 5% nonfat milk blocking suspension for 1 h, the membranes were incubated with antibodies against β-actin (1:1000), Bax (1:1000), Bcl-2 (1:1000), P-glycoprotein (1:1000), and LRP (1:25) at 4 °C overnight. HRP-conjugated secondary antibodies were incubated for 2 h with the membranes at a 1:5000 dilution. Immunore active bands were visualized using a Molecular Imager ChemiDoc XRS + System (Bio-Rad, Hercules, CA, USA) then analyzed using Image Lab™ Software (version 4.1, Bio-Rad, Hercules, CA, USA).

### 3.10. Statistical Analysis

Data were analyzed using GraphPad PrismV5.0 (GraphPad Software, Inc., San Diego, CA, USA). One-way ANOVA and Dunnett’s post hoc test were used to evaluate statistical significance. All data are expressed as mean ± SD. Statistical significance was set at *p* < 0.05 and *p* < 0.01.

## 4. Conclusions

These results demonstrate that the addition of JGGC can significantly increase the accumulation of HCPT in tissues, an increase which may be related to the inhibition of MDR-related protein. This may enhance the potential use of meridian transport effect drugs combined with an anticancer drug to better achieve active drug distribution. In addition, the considerable synergistic anticancer effect of JGGC may be attributed to the reduction in apoptotic resistance and improvement in immunity, and could provide a reference for the potential combined use of JGGC and HCPT in clinic.

## Figures and Tables

**Table 1:** Regression data of HCPT in all matrices.

	Linear Regression Equation	R	Linear Range (ng/mL)
Heart	y = 0.00664x + 0.0017	0.9980	1–500
Liver	y = 0.00519x + 0.0232	0.9983	5–2000
Spleen	y = 0.00681x + 0.00263	0.9995	1–500
Lung	y = 0.00653x + 0.0131	0.9978	1–500
Kidney	y = 0.00375x + 0.00666	0.9965	1–1000

**Table 2:** Precision, accuracy, and recovery of ultrahigh-performance liquid chromatography-tandem mass spectrometry (UHPLC-MS/MS) method for analysis of HCPT in tissue (n = 5).

Type of Matrics	Concentration (ng/mL)	Precision				Extraction Recovery (RSD, %)
		Intraday (RSD, %)	(RE, %)	Interday (RSD, %)	(RE, %)	
Heart	5	8.85	9.6	8.21	10.0	78.75 ± 5.39
	100	3.63	2.88	6.08	7.3	81.24 ± 3.38
	500	6.73	−1.00	4.58	5.90	86.05 ± 5.76
Liver	10	9.58	−6.3	7.79	1.3	77.44 ± 5.08
	100	5.7	8.76	4.63	4.03	83.21 ± 0.79
	1000	3.32	7.68	3.04	8.29	98.62 ± 2.04
Spleen	5	2.08	3.6	2.28	4.00	80.15 ± 4.92
	100	1.28	−3.45	1.07	9.68	81.34 ± 6.54
	500	4.73	1.02	4.78	−0.59	91.83 ± 4.33
Lung	5	4.32	11.2	7.04	9.6	83.31 ± 4.11
	100	6.65	7.16	6.72	4.79	97.99 ± 6.46
	500	6.78	5.27	5.06	7.32	101.44 ± 6.87
Kidney	5	3.34	−13.4	2.59	−6.00	84.62 ± 4.59
	100	5.87	7.79	6.86	10.43	92.08 ± 2.28
	500	4.38	−4.64	7.83	11.45	90.75 ± 2.80

**Table 3:** Stability of HCPT in tissue under various storage conditions (n = 5).

Type of Matric	Concentration (ng/mL)	Room Temperature for 8 h		Three Freeze-Thaw Cycles		−20 °C for 30 Days	
		RE (%)	RSD (%)	RE (%)	RSD (%)	RE (%)	RSD (%)
Heart	5	4.54	1.62	7.85	5.61	14.23	−6.07
	100	4.86	−12.32	5.69	3.99	5.21	−7.57
	500	0.47	−2.13	1.20	−0.98	13.67	−7.71
Liver	10	12.56	3.59	8.77	−1.31	4.92	8.33
	100	3.05	−3.16	0.56	−9.82	7.08	−7.12
	1000	3.50	−12.90	2.82	−10.84	6.31	0.76
Spleen	5	9.45	5.31	2.28	9.43	5.35	−3.63
	100	10.43	−2.44	7.78	−3.04	1.09	−13.70
	500	0.99	−13.55	6.66	−6.81	4.63	−1.47
Lung	5	6.30	−3.30	2.19	−0.87	3.84	−3.19
	100	8.01	5.48	2.06	−6.26	14.78	2.70
	500	9.10	9.85	11.76	−5.26	1.28	−13.45
Kidney	5	3.36	12.63	3.47	11.58	2.90	6.56
	100	11.77	5.88	10.81	1.79	2.29	0.75
	500	7.22	−1.64	3.30	10.09	0.06	6.94

**Table 4:** Mean pharmacokinetic parameters of HCPT in HCPT alone and HCPT combination with Platycodonis Radix-Glycyrrhizae Radix ET Rhizoma (JGGC).

Parameter	Heart		Liver		Spleen		Lung		Kidney	
	HCPT	JGGC-HCPT	HCPT	JGGC-HCPT	HCPT	JGGC-HCPT	HCPT	JGGC-HCPT	HCPT	JGGC-HCPT
AUC_(0–t)_ (ug/L·h)	34.31 ± 2.07	60.32 ± 10.97 *	5728.64 ± 988.35	8140.07 ± 899.51	167.50 ± 20.57	334.21 ± 39.85 **	143.29 ± 9.47	346.07 ± 56.31	398.35 ± 79.36	497.01 ± 54.23
C_max_ (µg/L)	21.32 ± 3.81	15.32 ± 1.28 *	2243.29 ± 567.07	2508.75 ± 460.75	99.66 ± 19.86	136.27 ± 18.06 *	66.48 ± 16.72	93.45 ± 16.22 *	280.30 ± 94.01	405.78 ± 133.80
T_1/2_ (h)	4.04 ± 1.37	3.65 ± 1.31	1.71 ± 0.19	1.73 ± 0.24	2.53 ± 0.55	3.95 ± 1.6	2.87 ± 0.75	2.97 ± 0.56	2.083 ± 0.74	2.073 ± 1.069
T_max_ (h)	0.5	0.5	0.5	1	0.5	0.6 ± 0.22	0.45 ± 0.11	0.6 ± 0.22	0.6 ± 0.224	0.45 ± 0.11
CLz (L/h/kg)	92.42 ± 12.57	53.18 ± 17.53 **	0.67 ± 0.11	0.46 ± 0.06	21.64 ± 3.20	9.07 ± 1.61 **	23.93 ± 2.57	9.85 ± 2.20 **	9.59 ± 1.79	7.39 ± 1.06
MRT_(0–t)_ (h)	3.04 ± 0.2	3.05 ± 0.17	2.48 ± 0.15	2.42 ± 0.10	2.12 ± 0.21	2.85 ± 0.08 *	2.34 ± 0.21	3.03 ± 0.19 **	2.18 ± 0.29	2.63 ± 1.03

AUC: area under the concentration-time curve; CL, clearance rate; MRT, mean residence time; T_max_, peak time; C_max_, peak concentration; T_1/2_, half-time. Data are presented as mean ± SD; * *p* < 0.05, ** *p* < 0.01 vs. HCPT.

**Table 5:** The relative organ weight (%) of HCPT alone and HCPT combination with JGGC.

Group	Heart	Liver	Spleen	Lung	Kidney
Normal	0.53 ± 0.05	3.96 ± 0.23	0.32 ± 0.04	0.70 ± 0.06	1.44 ± 0.06
Control	0.47 ± 0.07	4.38 ± 0.74 ^##^	0.68 ± 0.11 ^##^	0.68 ± 0.19	1.33 ± 0.12
HCPT	0.46 ± 0.04	4.37 ± 0.41 ^##^	0.83 ± 0.21 ^##^	0.63 ± 0.12	1.24 ± 0.07
L	0.46 ± 0.08	3.85 ± 0.36 **	0.83 ± 0.18 ^##^	0.61 ± 0.07	1.25 ± 0.09
M	0.46 ± 0.07	3.91 ± 0.33 **	0.81 ± 0.09 ^##^	0.59 ± 0.06	1.31 ± 0.08
H	0.45 ± 0.06	3.96 ± 0.37 **	0.79 ± 0.18 ^##^	0.63 ± 0.12	1.31 ± 0.11

Data are shown as mean ± SD, n = 10. ^#^ *p*< 0.05, ^##^ *p* < 0.01 vs. Normal; * *p* < 0.05, ** *p* < 0.01, vs. HCPT.
